# Supplementary material for: Extended-spectrum beta-lactamase (ESBL)-producing and non-ESBL-producing Escherichia coli isolates causing bacteremia in the Netherlands (2014 – 2016) differ in clonal distribution, antimicrobial resistance gene and virulence gene content
Source: PLoS One. 2020 Jan 14;15(1):e0227604. doi: 10.1371/journal.pone.0227604 (PMC6959556; doi:10.1371/journal.pone.0227604)
Supplement: S1 Appendix — (PDF) [file pone.0227604.s001.pdf]

EPIGENEC STUDY - SUPPORTING INFORMATION

Extended-spectrum beta-lactamase (ESBL)-producing and non-ESBL-producing *Escherichia coli* isolates causing bacteremia in the Netherlands (2014 – 2016) differ in clonal distribution, antimicrobial resistance gene and virulence gene content

Denise van Hout, Tess D. Verschuuren, Patricia C.J. Bruijning-Verhagen, Thijs Bosch, Anita C. Schürch, Rob J.L. Willems, Marc J.M. Bonten, Jan A.J.W. Kluytmans

S1 Appendix - content

S1A Figure. ST distribution among different onset of ECB

S1B Table. ST distribution among different onset of ECB

S1C Figure. ST distribution among different primary foci of ECB

S1D Table. ST distribution among different primary foci of ECB

S1A Figure. ST distribution among different onset of ECB[a]

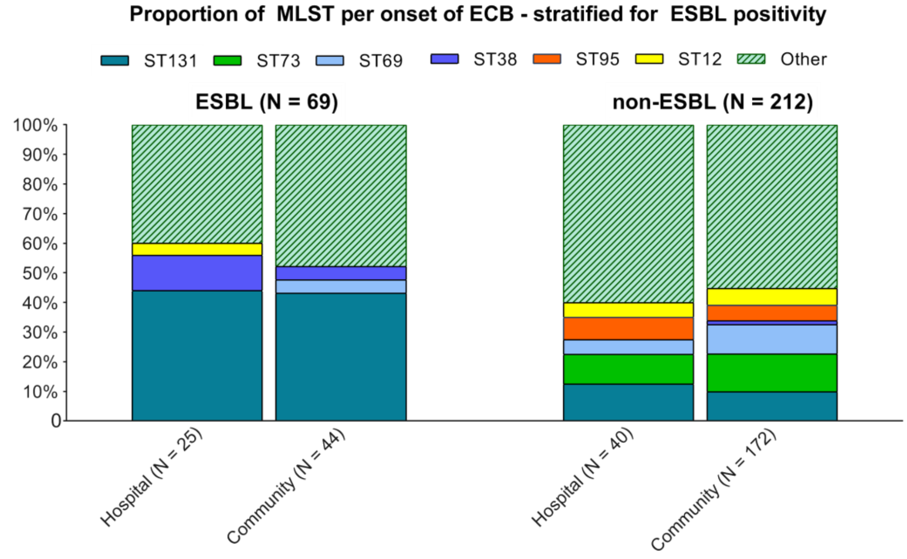

[a]ESBL-positivity based on phenotypic ESBL production.
ECB, *E. coli* bacteremia; ESBL, extended spectrum beta-lactamase; ST, sequence type.
Only STs that occurred >5% within non-ESBL-Ec or ESBL-Ec were grouped into main ST groups, the rest is categorized as "Other".

S2 Table. ST distribution among different onset of ECB

	**ESBL *E. coli*** [a]		**Non-ESBL *E. coli*** [a]	
	Hospital (N=25)	Community (N=44)	Hospital (N = 40)	Community (N = 172)
**ST131,** N (%)	11 (44)	19 (43)	5 (13)	17 (10)
**ST73,** N (%)	-	-	4 (10)	22 (13)
**ST69,** N (%)	-	2 (5)	2 (5)	17 (10)
**ST38,** N (%)	3 (12)	2 (5)	-	2 (1)
**ST95,** N (%)	-	-	3 (8)	9 (5)
**ST12,** N (%)	1 (4)	-	2 (5)	10 (6)
**Other ST,** N (%)	10 (40)	21 (48)	24 (60)	95 (55)

[a]ESBL-positivity based on phenotypic ESBL production.
ECB, *E. coli* bacteremia; ESBL, extended spectrum beta-lactamase; HB, hepatic-biliary; GI, gastro-intestinal; ST, sequence type

S3 Figure. ST distribution among different primary foci of ECB[a]

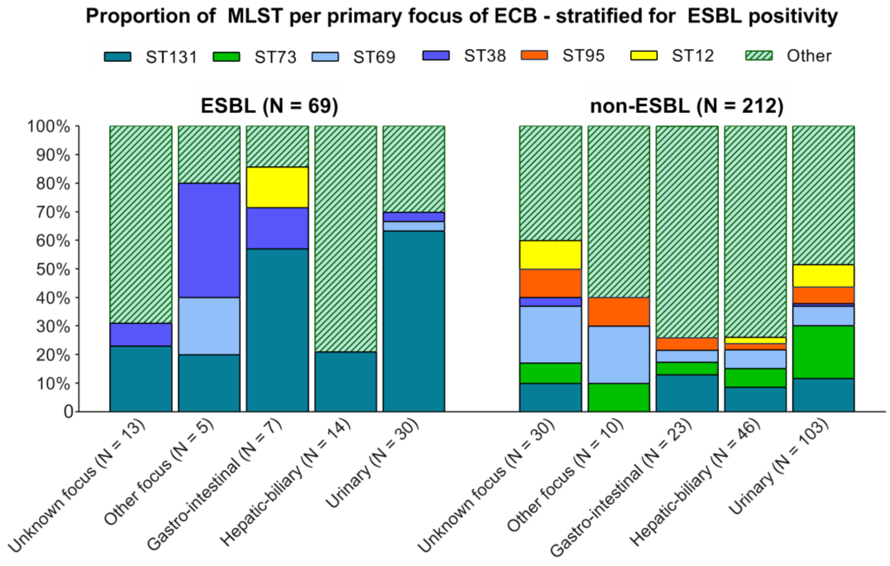

[a]ESBL-positivity based on phenotypic ESBL production.

ECB, *E. coli* bacteremia; ESBL, extended spectrum beta-lactamase; ST, sequence type

Only STs that occurred >5% within non-ESBL-Ec or ESBL-Ec were grouped into main ST groups, the rest is categorized as “Other”.

S4 Table. ST distribution among different primary foci of ECB[a]

	ESBL *E. coli*					Non-ESBL *E. coli*				
	Urinary (N=30)	HB (N=14)	GI (N=7)	Other (N=5)	Unknown (N=13)	Urinary (N=103)	HB (N=46)	GI (N=23)	Other (N=10)	Unknown (N=30)
**ST131,** N (%)	19 (63)	3 (21)	4 (57)	1 (20)	3 (23)	12 (12)	4 (9)	3 (13)	-	3 (10)
**ST73,** N (%)	-	-	-	-	-	19 (18)	3 (7)	1 (4)	1 (10)	2 (7)
**ST69,** N (%)	1 (3)	-	-	1 (20)	-	7 (7)	3 (7)	1 (4)	2 (20)	6 (20)
**ST38,** N (%)	1 (3)	-	1 (14)	2 (40)	1 (8)	1 (1)	-	-	-	1 (3)
**ST95,** N (%)	-	-	-	-	-	6 (6)	1 (2)	1 (4)	1 (10)	3 (10)
**ST12,** N (%)	-	-	1 (14)	-	-	8 (8)	1 (2)	-	-	3 (10)
**Other ST,** N (%)	9 (30)	11 (79)	1 (14)	1 (20)	9 (69)	50 (49)	34 (74)	17 (74)	6 (60)	12 (40)

[a]ESBL-positivity based on phenotypic ESBL production.

ECB, *E. coli* bacteremia; ESBL, extended spectrum beta-lactamase; HB, hepatic-biliary; GI, gastro-intestinal; ST, sequence type
