# Supplementary material for: Extended-spectrum beta-lactamase (ESBL)-producing and non-ESBL-producing Escherichia coli isolates causing bacteremia in the Netherlands (2014 – 2016) differ in clonal distribution, antimicrobial resistance gene and virulence gene content
Source: PLoS One. 2020 Jan 14;15(1):e0227604. doi: 10.1371/journal.pone.0227604 (PMC6959556; doi:10.1371/journal.pone.0227604)
Supplement: S2 Appendix — (PDF) [file pone.0227604.s002.pdf]

EPIGENEC STUDY - SUPPORTING INFORMATION

Extended-spectrum beta-lactamase (ESBL)-producing and non-ESBL-producing *Escherichia coli* isolates causing bacteremia in the Netherlands (2014 – 2016) differ in clonal distribution, antimicrobial resistance gene and virulence gene content

Denise van Hout, Tess D. Verschuuren, Patricia C.J. Bruijning-Verhagen, Thijs Bosch, Anita C. Schürch, Rob J.L. Willems, Marc J.M. Bonten, Jan A.J.W. Kluytmans

S2 Appendix

S2 Table. Frequencies of O:serotypes per primary focus of ECB

	ESBL *E. coli* (N = 69)[a]					Non-ESBL *E. coli* (N = 212)[a]				
	Urinary (N=30)	HB (N=14)	GI (N=7)	Other (N=5)	Unknown (N=13)	Urinary (N=103)	HB (N=46)	GI (N=23)	Other (N=10)	Unknown (N=30)
**O25,** N (%)	17 (57)	2 (14)	3 (43)	1 (20)	1 (8)	12 (12)	7 (15)	2 (9)	-	3 (10)
**O6,** N (%)	-	-	-	-	-	15 (15)	6 (13)	-	2 (20)	2 (7)
**O4,** N (%)	-	-	1 (14)	-	-	8 (8)	1 (2)	-	-	3 (10)
**O2/O50,** N (%)	-	-	-	-	-	10 (10)	6 (13)	-	1 (10)	2 (7)
**O75,** N (%)	-	-	-	-	-	4 (4)	1 (2)	2 (9)	1 (10)	3 (10)
**O1,** N (%)	1 (3)	-	-	-	-	2 (2)	1 (2)	-	-	1 (3)
**O8,** N (%)	-	4 (29)	-	-	-	5 (5)	3 (7)	6 (26)	2 (20)	1 (3)
**O15,** N (%)	1 (3)	-	-	1 (20)	-	5 (5)	-	-	-	4 (13)
**O18,** N (%)	-	-	-	-	-	5 (5)	-	-	1 (10)	1 (3)
**O16,** N (%)	2 (6)	1 (7)	1 (14)	-	-	2 (2)	-	2 (9)	-	-
**Other serotype**	9 (30)	7 (50)	2 (29)	3 (60)	12 (92)	35 (34)	21 (46)	11 (48)	3 (30)	10 (33)

[a]ESBL-positivity based on phenotypic ESBL production.

ESBL, extended spectrum beta-lactamase; HB, hepatic-biliary; GI, gastro-intestinal

Frequencies of all serotypes of the 4-valent and new potential 10-valent ExPEC vaccine are reported, the rest (including missing / unknown serotypes) is grouped as “Other serotype”. Percentages are column percentages.
