# Supplementary material for: Extended-spectrum beta-lactamase (ESBL)-producing and non-ESBL-producing Escherichia coli isolates causing bacteremia in the Netherlands (2014 – 2016) differ in clonal distribution, antimicrobial resistance gene and virulence gene content
Source: PLoS One. 2020 Jan 14;15(1):e0227604. doi: 10.1371/journal.pone.0227604 (PMC6959556; doi:10.1371/journal.pone.0227604)
Supplement: S3 Appendix — (PDF) [file pone.0227604.s003.pdf]

EPIGENEC STUDY - SUPPORTING INFORMATION

Extended-spectrum beta-lactamase (ESBL)-producing and non-ESBL-producing *Escherichia coli* isolates causing bacteremia in the Netherlands (2014 – 2016) differ in clonal distribution, antimicrobial resistance gene and virulence gene content

Denise van Hout, Tess D. Verschuuren, Patricia C.J. Bruijning-Verhagen, Thijs Bosch, Anita C. Schürch, Rob J.L. Willems, Marc J.M. Bonten, Jan A.J.W. Kluytmans

S3 Appendix - content

S3A Table. Detected acquired resistance genes with ResFinder 3.1.0 per antibiotic group

S3B Figure. Resistance gene count among epidemiological subgroups

S3C Table. Pairwise comparisons resistance gene score between epidemiological subgroups

S3D Table. Pairwise comparisons resistance gene score between STs

S3A Table. Detected resistance genes with ResFinder 3.1.0 per antibiotic group

(Broad-spectrum) Beta-lactamases			ESBL and ampC			Macrolides			Fluoroquinolones		
Gene	***N***	***(%)***	***Gene***	***N***	***(%)***	***Gene***	***N***	***(%)***	***Gene***	***N***	***(%)***
blaOXA-1	23	8%	*blaCMY-146*	1	0%	*ere(A)*	1	0%	*qnrA1*	2	1%
blaTEM-1A	8	3%	*blaCMY-2*	2	1%	*mph(A)*	48	17%	*qnrS1*	5	2%
blaTEM-1B	82	29%	*blaCTX-M-1*	6	2%	*mph(B)*	3	1%			
blaTEM-1C	8	3%	*blaCTX-M-102*	8	3%						
blaTEM-1D	2	1%	*blaCTX-M-14*	9	3%						
blaTEM-30	1	0%	*blaCTX-M-15*	29	10%						
blaTEM-34	1	0%	*blaCTX-M-27*	1	0%						
blaTEM-40	1	0%	*blaCTX-M-3*	1	0%						
			blaCTX-M-55	1	0%						
			blaCTX-M-9	2	1%						
			blaSHV-102	5	2%						
			blaSHV-12	1	0%						
			blaTEM-28	1	0%						
			blaTEM-35	1	0%						
			blaTEM-52B	1	0%						

Aminoglycosides			Sulfonamides and Trimetroprim			Tetracyclines			Other		
Gene	***N***	***(%)***	***Gene***	***N***	***(%)***	***Gene***	***N***	***(%)***	***Gene***	***N***	***(%)***
aac(3)-IIa	1	0%	*dfrA1*	18	6%	*tet(A)*	72	26%	*catA1*	12	4%
aac(3)-IIa	10	4%	*dfrA12*	6	2%	*tet(B)*	27	10%	*strA*	19	7%
aac(3)-IId	4	1%	*dfrA14*	10	4%	*tet(D)*	1	0%	*strB*	10	4%
aac(3)-IId	7	2%	*dfrA17*	44	16%	*tet(J)*	1	0%	*cat*	1	0%
aac(3)-Iva	1	0%	*dfrA21*	1	0%	*tet(M)*	1	0%	*cmlA1*	6	2%
aac(3)-Via	1	0%	*dfrA5*	12	4%	*tet(X)*	1	0%	*mdf(A)*	260	93%
aac(6')-Ib-cr	12	4%	*dfrA7*	9	3%				*floR*	8	3%
aac(6')Ib-cr	8	3%	*dfrA8*	2	1%				*lnu(F)*	5	2%
aadA1	11	4%	*sul1*	64	23%						
aadA2	10	4%	*sul2*	86	31%						
aadA4	1	0%	*sul3*	6	2%						
aadA5	39	14%									
ant(2")-Ia	4	1%									
ant(3")-Ia	22	8%									
aph(3")-Ib	63	22%									
aph(3')-Ia	24	9%									
aph(3')-Ib	1	0%									
aph(4)-Ia	1	0%									
aph(6)-Id	69	25%									

In case genes were present twice within a strain, they were only counted once in the resistance gene count.

S3B Figure. Acquired resistance gene count among epidemiological subgroups

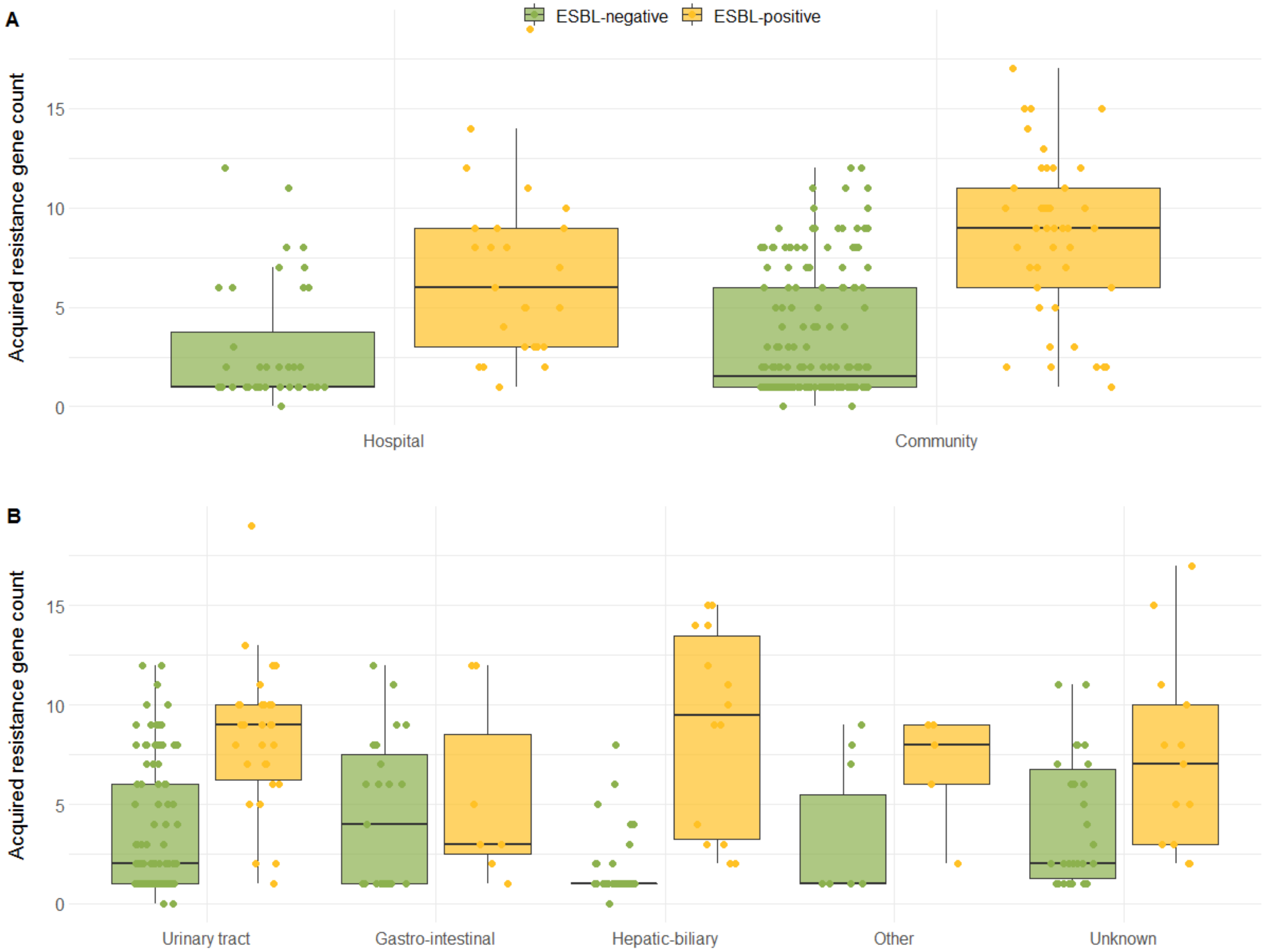

ESBL, extended-spectrum beta-lactamase. ESBL-positivity was based on phenotypic ESBL-production.
Boxplots display median and inter quartile range and every dot represents a single isolate. The ResFinder 3.1.0 database was used to determine acquired resistance genes. **A**. Resistance gene count per onset of infection, stratified for non-ESBL-Ec and ESBL-Ec isolates. **B**. Resistance gene count per primary focus of ECB, stratified for non-ESBL-Ec and ESBL-Ec isolates

S3C Table. Pairwise comparisons acquired resistance gene count between epidemiological subgroups

	Median resistance gene count (IQR)				Pairwise comparisons between groups, within non-ESBL and ESBL[a]	
	**Non-ESBL**	**ESBL**			**Non-ESBL**	**ESBL**
**Onset of infection**			**Group 1**	**Group 2**		
Community (N = 216)	2 (1–6)	9 (6–11)	Community	Hospital	NS[b]	NS
Hospital (N = 65)	1 (1–4)	6 (3–9)				
**Primary focus**			**Group 1**	**Group 2**		
Urinary tract (N = 133)	2 (1–6)	9 (7–10)	Urinary	GI	NS	NS
Hepatic- biliary (N = 60)	1 (1–1)	10 (3–14)	Urinary	HB	2.8e-04***	NS
Gastro-intestinal (N = 30)	4 (1–8)	3 (3–9)	Urinary	Other	NS	NS
Unknown (N = 43)	2 (1–7)	7 (3–10)	Urinary	Unknown	NS	NS
Other (N = 15)	1 (1–6)	8 (6–9)	GI	HB	5.8e-03**	NS
			GI	Other	NS	NS
			GI	Unknown	NS	NS
			HB	Other	NS	NS
			HB	Unknown	3.1e-05****	NS
			Other	Unknown	NS	NS

ESBL, extended-spectrum beta-lactamase; HB, hepatic-biliary; GI, gastro-intestinal; IQR, interquartile range; NA, not applicable; NS, not significant

[a] Groups were compared with Wilcoxon rank sum Test and *P* values were adjusted with the Holm-Bonferroni correction to adjust for multiple testing. ESBL-positivity was based on phenotypic ESBL-production.

[b] *P* value represents the adjusted *P* value for the comparison of the resistance gene count of Group 1 versus Group 2, within the non-ESBLs or ESBLs (i.e. *P* value 2.8e-04 is the *P* value for the comparison in acquired resistance gene count in urinary versus hepatic-biliary primary focus among non-ESBL *E. coli*)

*, **, *** and **** indicate *P* values ≤0.05, ≤0.01, ≤0.001 and ≤0.0001.

The ResFinder 3.1.0 database was used to determine presence of acquired resistances genes. Gene counts were rounded to whole numbers if applicable.

S3D Table. Pairwise comparisons acquired resistance gene count between dominant STs[a]

	Median resistance gene count (IQR)				Pairwise comparisons between groups, within non-ESBL and ESBL[b]	
	Non-ESBL	ESBL			Non-ESBL	ESBL
			Group 1	Group 2		
Other ST (N = 150)	1 (1–6)	8 (3–12)	ST12	ST131	NS	NS
ST131 (N = 52)	2 (1–5)	9 (6–10)	ST12	ST38	NS	NS
ST73 (N = 26)	1 (1–2)	NA	ST12	ST69	NS	NS
ST69 (N = 21)	6 (1–8)	9 (9–9)	ST12	ST73	NS	NS
ST12 (N = 13)	1 (1–3)	3 (3–3)	ST12	ST95	NS	NS
ST95 (N = 12)	1 (1–2)	NA	ST131	ST38	NS	NS
ST38 (N = 7)	8 (7–8)	5 (5–8)	ST131	ST69	NS	NS
			ST131	ST73	NS	NS
			ST131	ST95	NS	NS
			ST38	ST69	NS	NS
			ST38	ST73	NS	NS
			ST38	ST95	NS	NS
			ST69	ST73	NS	NS
			ST69	ST95	NS	NS
			ST73	ST95	NS	NS

ESBL, extended-spectrum beta-lactamase; NA, not applicable; NS, not significant; ST, sequence type
ESBL-positivity was based on phenotypic ESBL-production.
[a] Comparisons with category “Other” are not shown; because of heterogeneity in STs this comparison was not considered as informative.
[b] Pairwise comparisons were made with Wilcoxon rank sum Test and *P* values were adjusted with the Holm-Bonferroni correction to adjust for multiple testing.
The ResFinder 3.1.0 database was used for determination of acquired resistance genes. Gene counts were rounded to whole numbers if applicable.
