# Supplementary material for: Extended-spectrum beta-lactamase (ESBL)-producing and non-ESBL-producing Escherichia coli isolates causing bacteremia in the Netherlands (2014 – 2016) differ in clonal distribution, antimicrobial resistance gene and virulence gene content
Source: PLoS One. 2020 Jan 14;15(1):e0227604. doi: 10.1371/journal.pone.0227604 (PMC6959556; doi:10.1371/journal.pone.0227604)
Supplement: S4 Appendix — (PDF) [file pone.0227604.s004.pdf]

EPIGENEC STUDY - SUPPORTING INFORMATION

Extended-spectrum beta-lactamase (ESBL)-producing and non-ESBL-producing *Escherichia coli* isolates causing bacteremia in the Netherlands (2014 – 2016) differ in clonal distribution, antimicrobial resistance gene and virulence gene content

Denise van Hout, Tess D. Verschuuren, Patricia C.J. Bruijning-Verhagen, Thijs Bosch, Anita C. Schürch, Rob J.L. Willems, Marc J.M. Bonten, Jan A.J.W. Kluytmans

<u>S4 Appendix - content</u>

S4A Table. Detected ExPEC-associated VG per VG category

S4B Figure. VG count among epidemiological subgroups

S4C Table. Pairwise comparisons VG score between epidemiological subgroups

S4D Table. Pairwise comparisons VG score between STs

S4A Table. Detected ExPEC-associated VG per VG category[a]

Adhesins		Siderophores		Protectins and invasins		Toxins		Other	
**Gene**	***N (%)[b]***	**Gene**	***N (%)[b]***	**Gene**	***N (%)[b]***	**Gene**	***N (%)[b]***		***N (%)[b]***
yagZ/ecpA	271 (96.4)	*sitA*	233 (82.9)	*ompA*	235 (83.6)	*usp*	158 (56.2)	*traT*	181 (64.4)
fimH	266 (94.7)	*fyuA*	224 (79.7)	*ompT*	218 (77.6)	*vat*	101 (35.9)	*malX*	164 (58.4)
tia	124 (44.1)	*chuA*	158 (56.2)	*kpsM*[c]	78 (27.8)	*sat*	91 (32.4)	*iss*	124 (44.1)
iha	111 (39.5)	*iroN*	135 (48.0)	*tcpC*	53 (18.9)	*clbB*	80 (28.5)	*cvaC*	42 (14.9)
papC	103 (36.7)	*iutA*	32 (11.4)	*ibeA*	40 (14.2)	*clbN*	80 (28.5)	*fliC*	19 (6.8)
papH	100 (35.6)	*ireA*	39 (13.9)			*hlyD*	76 (27.0)	*rfc*	13 (4.6)
sfa/foc[c]	87 (40.1)					*hlyA*	72 (25.6)		
agn43	81 (28.8)					*cnf1*	66 (23.5)		
papG	57 (20.3)					*pic*	45 (16.0)		
papF	55 (19.6)					*astA*	29 (10.3)		
afa/dra[c]	43 (15.3)					*cdtB*	13 (4.6)		
nfaE	9 (3.2)								
gafD	8 (2.8)								
bmaE	7 (2.5)								
papE	7 (2.5)								
papA	5 (1.8)								

VG, virulence genes

[a] The following genes were not detected in any of the isolates: *focE, hra, yfcV* and *tsh* (adhesins) and *hlyF* (toxin)

[b] N indicates numbers of isolates with gene, % of all isolates (N = 281)

[c] The *kpsM*, *afa/dra* and *sfa/foc* operons were considered present if any of the corresponding genes our allelic variants were identified.

S4B Figure. ExPEC-associated VG score in different subgroups, stratified for ESBL-positivity[a]

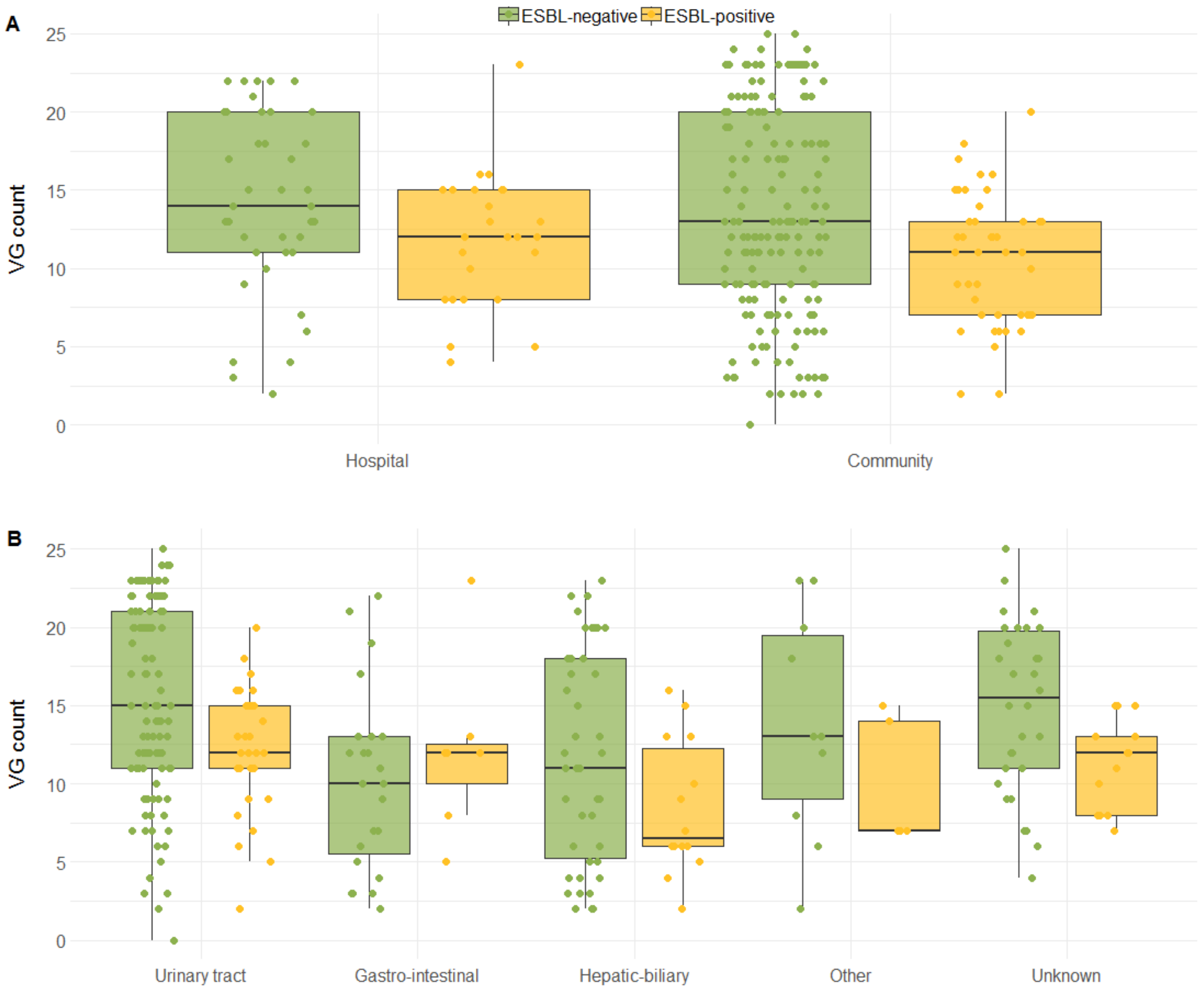

ESBL, extended spectrum beta-lactamase; VG, virulence genes.

[a]ESBL-positivity was based on phenotypic ESBL-production.

Boxplots display median and inter quartile range (IQR) and every dot represents a single isolate. **A**. VG count per onset of infection, stratified for non-ESBL-Ec and ESBL-Ec isolates. **B**. VG count per primary focus of ECB, stratified for non-ESBL-Ec and ESBL-Ec isolates.

S4C Table. Pairwise comparisons VG score between epidemiological subgroups

	Median VG score (IQR)		Pairwise comparisons between groups, within non-ESBL and ESBL[a]			
**Onset of infection**	**Non-ESBL**	**ESBL**	**Group 1**	**Group 2**	**Non-ESBL**	**ESBL**
Community (N = 216)	13 (9–20)	11 (7–13)	Community	Hospital	NS	NS
Hospital (N = 65)	14 (11–20)	12 (8–15)				
**Primary focus**			**Group 1**	**Group 2**		
Urinary tract (N = 133)	15 (11–21)	12 (11–15)	Urinary	GI	0.0072**	NS
Hepatic- biliary (N = 60)	11 (5–18)	7 (6–13)	Urinary	HB	0.036*	NS
Gastro-intestinal (N = 30)	10 (5–13)	12 (8–13)	Urinary	Other	NS	NS
Unknown (N = 43)	16 (11–20)	12 (8–13)	Urinary	Unknown	NS	NS
Other (N = 15)	13 (8–20)	7 (7-14)	GI	HB	NS	NS
			GI	Other	NS	NS
			GI	Unknown	NS	NS
			HB	Other	NS	NS
			HB	Unknown	NS	NS
			Other	Unknown	NS	NS
**Urinary catheter**			**Group 1**	**Group 2**		
No (N = 184)	13 (9-20)	10 (7-13)	No catheter	Catheter	NS	NS
Yes (N = 97)	13 (7-18)	13 (11-15)				
**30-day mortality**			**Group 1**	**Group 2**		
Alive (N = 238)	13 (9-20)	12 (8-15)	Alive	Deceased	NS	NS
Deceased (N = 43)	12 (6-18)	11 (7-14)				
**Admission ward**			**Group 1**	**Group 2**		
Non-ICU (N = 240)	13 (9-20)	12 (7-15)	Non-ICU	ICU	NS	NS
ICU (N =41)	13 (6-18)	12 (10-14)				

ESBL, expended-spectrum beta-lactamase; HB, hepatic-biliary; GI, gastro-intestinal; IQR, interquartile range; NA, not applicable; NS, not significant; VG, virulence gene.
[a] Pairwise comparisons were made with Wilcoxon rank sum Test and *P* values were adjusted with the Holm-Bonferroni correction to adjust for multiple testing.
* and ** indicate *P* values ≤0.05 and ≤0.01. Gene counts were rounded to whole numbers if applicable. ESBL-positivity was based on phenotypic ESBL-production.

S14D Table. Pairwise comparisons VG score between dominant STs[a]

	Median VG score (IQR)		Pairwise comparisons between groups within non-ESBL and ESBL[b]			
	Non-ESBL	ESBL	Group 1	Group 2	Non-ESBL	ESBL
Other ST (N = 150)	11 (7–17)	8 (6 –11)	ST12	ST131	3.2e-05****	NS
ST131 (N = 52)	13 (12–15)	13 (12–15)	ST12	ST38	NS	NS
ST73 (N = 26)	22 (20–23)	-	ST12	ST69	5.5e-05****	NS
ST69 (N = 21)	11 (9–12)	8 (7–8)	ST12	ST73	NS	-
ST12 (N = 13)	22 (21–23)	23 (23–23)	ST12	ST95	0.032*	-
ST95 (N = 12)	18 (17–19)	-	ST131	ST38	NS	NS
ST38 (N = 7)	7 (6–7)	8 (7–8)	ST131	ST69	4.4e-03**	NS
			ST131	ST73	1.9e-07****	-
			ST131	ST95	41.9e-03**	-
			ST38	ST69	NS	NS
			ST38	ST73	NS	-
			ST38	ST95	NS	-
			ST69	ST73	2.0e-07****	-
			ST69	ST95	5.8e-05****	-
			ST73	ST95	0.032*	-

ESBL, extended-spectrum beta-lactamase; NS, not significant; ST, sequence type; VG, virulence gene.
[a] Comparisons with category “Other” are not shown; because of heterogeneity in STs this comparison is not considered as informative.
[b] Groups were compared with Wilcoxon rank sum Test and P-values were adjusted with the Holm-Bonferroni correction to adjust for multiple testing. ESBL-positivity was based on phenotypic ESBL-production.
*, **, *** and **** indicate P-values ≤0.05, ≤0.01, ≤0.001 and ≤0.0001.
Gene counts were rounded to whole numbers if applicable.
