# Supplementary material for: Extended-spectrum beta-lactamase (ESBL)-producing and non-ESBL-producing Escherichia coli isolates causing bacteremia in the Netherlands (2014 – 2016) differ in clonal distribution, antimicrobial resistance gene and virulence gene content
Source: PLoS One. 2020 Jan 14;15(1):e0227604. doi: 10.1371/journal.pone.0227604 (PMC6959556; doi:10.1371/journal.pone.0227604)
Supplement: Attachment — Submitted filename: Response to reviewers.pdf [file pone.0227604.s005.pdf]

**Date**: December 16, 2019
Utrecht, The Netherlands

**Re: PONE-D-19-29528 entitled " Extended-spectrum beta-lactamase (ESBL)-producing and non-ESBL-producing Escherichia coli isolates causing bacteremia in the Netherlands (2014 – 2016) differ in clonal distribution, antimicrobial resistance gene and virulence gene content"**

## Response to Reviewer #1

*This is a well-designed, and overall well performed study. Lots of data that have been obtained here, and Tables and Figures and well thought in order to make the whole set easy to understand and analyse.*

**Response**: We would sincerely like to thank the reviewer for the time taken to critically appraise this manuscript and the input to further improve the manuscript. In the following we will address the comments point by point.

*I would suggest to include S1, S2 and S3 in the main manuscript rather than as Supplements, but this is a detail somehow.*

**Response**: Thank you for this suggestion. We have indeed considered to move some of the figures from the Supplement in to the main manuscript, given they provide additional illustrative information. However, given the manuscript is very data-dense, including 4 figures and 2 tables already, we are afraid addition of more figures might be a bit too much. We would therefore like to request the reviewer to keep the figures in the supplementary material.

*What is missing on my opinion to provide the reader all the tools to analyse those data in term of population structure distribution are some words about the links between ST. Indeed it is essential to figure out whether a so-called ST73 for instance is phylogenetically close or by contrast very different from an ST131. This would mean completely different things as you may guess.*

**Reply**: Thank you for this comment. We indeed agree with the reviewer that the manuscript indeed lacked guidance on how to interpret the results of the different ST distributions. We have therefore added a comment to the Methods section that includes a short explanation of the MLST scheme (lines 101-102 of the revised manuscript) and have added a short comment to the Discussion section about the known phylogenetic relationship between ST73 and ST131, see lines 238-239 of the revised manuscript.

*The part dealing with virulence factors might be very much reduced on my opinion. In fact, the entire manuscript would benefit from signifiant shortening, and to condense it would surely make it more attractive to the reader.*

Julius Center for Health Sciences and Primary Care

**Epidemiology of Infectious Diseases**

Denise van Hout, MD
PhD Candidate

T: +31 88 75 67407
E: D.vanhout-3@umcutrecht.nl

Visiting address:
Universiteitsweg 100
3584 CG Utrecht

Postal address:
Internal mail no 6.131
P.O. Box 85500
3508 GA Utrecht

**www.umcutrecht.nl**

**Reply**: We agree with the reviewer that the part on virulence genes was too elaborate and have therefore shortened this paragraph by almost half (lines 209-216 of the revised manuscript). Furthermore, the rest of the manuscript was also shortened (by 20%), as suggested by the reviewer and the academic editor (mainly in the introduction, methods and in the description of results on resistance gene and VG content).

**Response to Reviewer #2**

*The manuscript describes a molecular and serologic epidemiological study of E. coli isolates from bacteremia ESBL and non- ESBL producers. The manuscript is well written, and despite not showing results completelly new, is interesting to a scientific reader and add more data on the epidemiology of E. coli recovered from blood samples.*
*The molecular study is very complete and WGS was used for analysing the genome of the isolates.*

**Reply**: We would like to thank the reviewer for the time and we sincerely appreciate the careful reading of our manuscript. In the following we respond to the comments point by point.

*I just have a few remarks:*
*Line 276: some VG scores were similar, not completelly different (lines 250-59)*

**Reply**: Thank you for this comment. The reviewer is right to point out that when assessing epidemiological subgroups, most of the VG scores were not significantly different between groups. These analyses were stratified on ESBL-positivity, thus were performed for non-ESBL-Ec and ESBL-Ec separately. We would like to point out that the statement made in line 276 however (line 233 of the revised manuscript), does not refer to the epidemiological subgroups, but refers to the overall significant difference in VG score that was found between non-ESBL-Ec and ESBL-Ec isolates, as provided in lines 211-212 of the revised manuscript. The paragraph on VG scores has been shortened and hopefully is now more clear to the reader.

*Line 309: It is well known that usually in E. coli there is an inversion in resistance genes versus virulence genes, mostly in UPEC (see Johnson et al papers). The exception is ST131, the most well studied ST right now. So, this is not a novelty.*

**Reply**: We thank the reviewer for this comment; we agree that the paragraph did not appropriately reflect current literature and have revised it accordingly. This also included referencing of additional papers, among which important papers performed by Johnson et al., Vila et al. and Velasco et al. (lines 267-273 of the revised manuscript).

*Minor comment: Introduction, authors repeat consecutively references (1-4) from lines 62 until 69. Not necessary.*

**Reply**: Thank you for this suggestion, the changed were made as suggested by the reviewer.
